# Shedding Light on Host-to-Yb^3+^ Energy Transfer in Cs_2_AgBiBr_6_:Yb^3+^ (nano)crystals

**DOI:** 10.1021/acs.chemmater.3c03201

**Published:** 2024-03-04

**Authors:** Jur W. de Wit, Lars L. Sonneveld, Andries Meijerink

**Affiliations:** Debye Institute for Nanomaterials Science, Utrecht University, Utrecht 3584 CC, The Netherlands

## Abstract

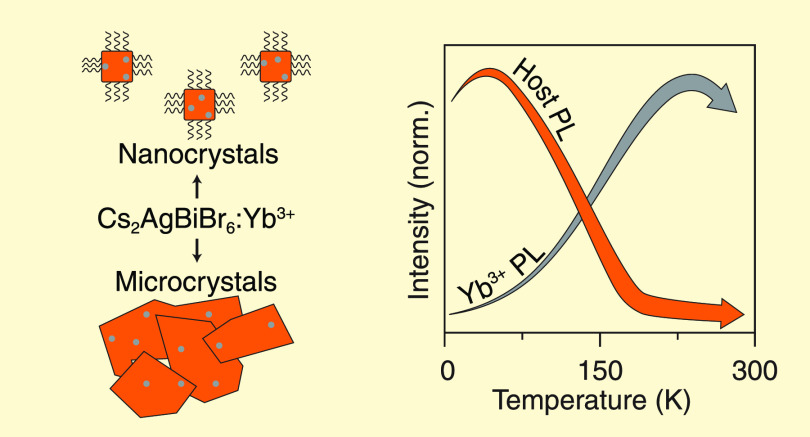

The optical properties of Cs_2_AgBiBr_6_ double perovskite nanocrystals have attracted considerable attention as lead-free alternatives to lead halide perovskites. A promising strategy to create additional flexibility in the emission color is doping lanthanide ions into Cs_2_AgBiBr_6_. Incorporating Yb^3+^ in the lattice has been shown to give rise to near-infrared (NIR) emission, but the energy transfer mechanism remained unclear. Here, we report on the luminescence and sensitization mechanism of Yb^3+^ in Cs_2_AgBiBr_6_ nano- and microcrystals. We observe that the incorporation of Yb^3+^ in the host lattice does not strongly affect the broadband red emission of the Cs_2_AgBiBr_6_ host but does give rise to an additional and characteristic ∼1000 nm NIR line emission from Yb^3+^. Temperature-dependent and time-resolved photoluminescence studies of undoped and Yb-doped Cs_2_AgBiBr_6_ reveal that the energy transfer does not take place through the red emissive state of the Cs_2_AgBiBr_6_ host. Instead, there is a competition between relaxation to the red-emitting state and trapping of the photoexcited charge carriers on Yb^3+^. Trapping on Yb^3+^ subsequently results in a charge transfer state that relaxes to the ^2^F_5/2_ excited state of Yb^3+^, followed by NIR narrow line f–f emission to the ^2^F_7/2_ ground state.

## Introduction

Lanthanide-doped nanocrystals (NCs) have found diverse applications because of their efficient and narrow line emission across the UV, visible, and (N)IR spectral ranges. Due to the shielding provided by the 5s- and 5p orbitals, the energy levels of 4f–4f transitions remain unaffected by the local coordination and give rise to characteristic luminescence properties. Narrow line emission is of particular interest, for example, for color conversion phosphors in blue LED-based displays where a high color purity helps to expand the color gamut. However, a disadvantage of lanthanides is that intraconfigurational f–f transitions are forbidden according to the parity selection rule and, therefore, absorb light weakly. Many applications of phosphors, however, require strong broadband absorption. To address this issue, a stronger absorbing species is typically introduced to transfer its energy nonradiatively to the emitting lanthanide ion, which is known as sensitization. Traditionally, strongly absorbing luminescent ions such as Ce^3+^ or Eu^2+^ have been codoped as sensitizers of lanthanide ions showing the characteristic f–f line emission of the desired color.

A promising alternative approach is the use of semiconductor nanocrystals (NCs) as a sensitizer for lanthanide luminescence and combining their strong, broadband, and size-tunable absorption with the desired lanthanide line emission. In recent years, lanthanides have been doped in several semiconductor NC systems, such as CdSe^1^ and InP/YF_3_ core/shell^2^ and PbIn_2_S_4_ NCs.^3^ However, it is difficult to incorporate large, trivalent lanthanide ions in lattices that only have cation sites with a coordination number (CN) of 4, which is the case for the traditional II/VI and III/V colloidal quantum dots having wurtzite- or zincblende-type structures, as lanthanides require CNs of 6 or higher. The doping of Yb^3+^ in the perovskite CsPbCl_3_ NCs on Pb^2+^ sites with CN = 6 has been successful and resulted in spectacular near-infrared (NIR) quantum yields (QYs) of almost 200%, caused by a quantum cutting process.^4,5^ Research efforts directed at incorporation of other lanthanides in CsPbCl_3_ have emerged and demonstrated incorporation of both Er^3+^ and Yb^3+^.^6^ Claims for the incorporation of many other lanthanides (including Ce^3+^, Sm^3+^, Eu^3+^, Tb^3+^, and Dy^3+^) have also been made but have so far been difficult to reproduce.^7^ More recently, elpasolite (or double perovskite) NCs have emerged as a promising host material. In this host, two divalent ions of the perovskite are replaced by a monovalent and trivalent ion arranged in an ordered manner. This offers the possibility of replacing the large six-coordinated trivalent ion with a trivalent lanthanide and allows for doping without charge compensation and offers a new family of hosts for semiconductor-to-Ln^3+^ energy transfer (ET).^8^ The cubic elpasolite structure is of the A_2_^+^M^+^M^3+^X_6_^–^ form, with Cs_2_AgBiBr_6_ as a well-studied workhorse material.^9^ Similar to perovskite NCs, the optical properties can be adjusted by changing their chemical composition while also being free of toxic lead.^10^ The class of materials is not novel. In the 1970s, insulator elpasolite microcrystals (MCs) and single crystals became notable hosts for luminescent lanthanide ions because of their highly symmetrical trivalent cation site. Richardson’s group, among others, utilized the nearly perfect octahedral symmetry of Cs_2_NaYCl_6_: Ln^3+^, a wide bandgap insulator material, for gaining theoretical understanding of intraconfigurational lanthanide transitions.^11,12^ More recently, doping various lanthanide ions into different elpasolite NCs has opened up many new prospects for sensitizing lanthanide emission with the absorption of semiconductor NCs.^13^

There is ongoing debate about the ET process from semiconductor host NCs to lanthanide dopants. In perovskite CsPbCl_3_ NCs doped with Yb^3+^, QYs close to 200% have been achieved through quantum cutting.^4^ Here, the mechanism involves the formation of a trapped exciton state at the lattice distortion, where the two Yb^3+^ ions replace three Pb^2+^ ions. Cooperative ET from the exciton state excites both Yb^3+^ ions and explains the almost 200% QY.^14,15^ In the case of Cs_2_(Ag,Na)(In,Bi)(Cl,Br)_6_ micro- and nanocrystals, the results are less conclusive. There is evidence that Bi codoping is required to enable ET from the Cs_2_A_g1-x_Na_*x*_InCl_6_ elpasolite host to various lanthanides.^16,17^ Photoluminescence excitation measurements of NIR emitting Er^3+^ in these crystals confirm that the lanthanide luminescence is sensitized via Bi^3+1^S_0_ → ^3^P_0, 1_ transitions which involve localized transitions in this host.^18^ Excitation measurements on Cs_2_AgBiBr_6_ NCs doped with Yb^3+^ and Mn^2+^ show that both host and lanthanide emission are sensitized by the excitonic absorption band resulting from delocalized conduction band (CB) states (which still have considerable Bi^3+^ 6p character^19^).^20^ For Yb^3+^-doped double perovskites, a mechanism similar to that for Yb-doped perovskites has been proposed where ET occurs from the red-emitting self-trapped exciton (STE) state to (a single) Yb^3+^ ion.^21^ Alternatively, direct ET from the host to Yb^3+^ has been depicted.^22^ In recent research on Cs_2_AgBiBr_6_:Yb^3+^, very high NIR QYs of more than 80% were reported for thin films fabricated with physical vapor deposition.^22,23^ For these Yb-doped thin films, the sensitization mechanism is not discussed in detail. In an earlier study on thin film Cs_2_AgBiBr_6_:Yb^3+^ based on room temperature spectroscopy and DFT calculations, the proposed ET mechanism involves the trapping of a CB electron by Yb^3+^ followed by the release of the electron to the CB, leaving Yb^3+^ behind in the excited state.^24^ To gain deeper insights into the host-to-Yb^3+^ ET mechanism, here, we report temperature-dependent and time-resolved spectroscopy studies on Yb^3+^-doped Cs_2_AgBiBr_6_. Previous variable-temperature studies of undoped Cs_2_AgBiBr_6_ have shown that the broad red host emission experiences strong thermal quenching at room temperature. Hence, temperature-dependent emission and time-resolved emission measurements could aid in differentiating between different ET mechanisms.

We synthesized both undoped and Yb^3+^-doped Cs_2_AgBiBr_6_ NCs and MCs and conducted temperature-dependent emission and time-resolved emission spectroscopy measurements down to 4 K. The Yb incorporation is monitored with luminescence spectroscopy and inductively coupled plasma atomic emission spectroscopy (ICP-OES) measurements. Interestingly, while the red host emission shows its previously reported thermal quenching, the Yb emission intensity increases with increasing temperature. This shows that direct ET from the red-emitting host state to Yb^3+^ is not the operative transfer mechanism. Based on our findings, we propose that a photoexcited electron–hole pair can either relax to the self-trapped state responsible for the red host emission or, and in competition with the former, the electron can localize on Yb^3+^, thereby reducing it to Yb^2+^. Subsequent capturing of the photogenerated hole by Yb^2+^ results in the formation of Yb^3+^ in the excited ^2^F_5/2_ state through a charge transfer (CT) state. The interesting increase in the Yb^3+^ emission intensity with temperature is a result of the increased hole mobility. At elevated temperatures, the hole mobility increases the probability for relaxation via electron and hole capture by Yb^2+^,^25^ resulting in higher Yb^3+^ emission intensity.

## Experimental Section

### Synthesis

The Cs_2_AgBiBr_6_ NCs were synthesized using the hot injection method based on the publication of Creutz et al.^26^ Typically, Cs_2_CO_3_ (0.355 mmol), CH_3_COOAg (0.5 mmol), (CH_3_CO_2_)_3_Bi (0.5 mmol), oleylamine (0.5 g), oleic acid (2.5 g), and octadecene (10 mL) were added to a 50 mL 3-neck flask connected to a Schlenk line. The solution was then degassed for 45 min under vacuum. During degassing, the solution turned from colorless to yellow to dark brown. Then, the temperature was increased to 145 °C under a nitrogen atmosphere, and TMS-Br (0.34 mL) was swiftly injected into the reaction mixture under vigorous stirring. A yellow precipitate immediately formed. After 15 s, the reaction vessel was submerged in an ice-water bath to quench the reaction. The cooled mixture was collected and centrifuged for 10 min at 4000 rpm (RCF = 3112 g). The dark brown supernatant was thoroughly drained, and the yellow precipitate was dispersed in toluene (5 mL) with sonication (10 min). The solution was centrifuged again for 10 min at 4000 rpm. The orange supernatant containing the NCs was collected for further characterization. Doping the Cs_2_AgBiBr_6_ NCs with Yb^3+^ was done by adding 0.025 mmol of Yb(CH_3_CO_2_)xH_2_O to the reaction mixture.

The Cs_2_AgBiBr_6_ MCs were synthesized based on a protocol for Cs_2_AgInCl_6_ MCs.^27^ The MCs were typically prepared by combining BiBr_3_ (0.5 mmol), AgBr (0.5 mmol), and YbCl_6_ · 6H_2_O (0.2 mmol) and dissolving this in 4 mL of HBr (9 M).^27^ The solution was heated to ∼70 °C until complete dissolution. Then 1 mmol CsBr was added to the solution, after which an orange precipitate immediately formed. This solution was heated for 20 more minutes before it was decanted. The powder was washed twice with ethanol and dried in an oven (70 °C) before being stored in a nitrogen-filled glovebox.

### Characterization

XRD measurements were performed on a Panalytical Aeris diffractometer with Cu K_alpha_ radiation at 40 kV. The NC measurements were done by drop-casting a NC solution on a low-background Si-wafer until a thin film had formed. Measurements on MCs were performed by placing the powder on a solid-state sample holder. TEM images were taken on a FEI T120C 100 keV microscope by drop-casting dilute NC solutions on a carbon-coated copper grid. The elemental analysis was carried out with a PerkinElmer ICP-OES (Optima 8300) after the NCs were completely dissolved in concentrated (65%) nitric acid. To ensure accurate measurements of only incorporated Yb and not surface or dissolved Yb, the NCs were washed with acetonitrile prior to the measurement.

Samples for optical characterization were prepared by diluting the nanocrystal stock solution 300 times (300 dilutions yield an absorbance of 0.07 at 430 nm) in toluene in a 10 × 10 mm quartz cuvette. Absorption spectra were measured on a PerkinElmer Lambda 950 UV/vis/IR spectrometer. Photoluminescence emission and excitation spectra on both NCs and MCs were measured on an Edinburgh Instruments FS920 spectrometer with a 450 W xenon light source. Luminescence spectra in the 400–850 nm range were recorded using a Hamamatsu R928 photomultiplier tube, while 800–1600 nm spectra were recorded using a liquid nitrogen-cooled Hamamatsu R5509 photomultiplier tube. For the optical measurements on NCs, a quartz cuvette was used, while for the MCs, a thin layer of powder was mounted in a holder. Room-temperature photoluminescence decay measurements were performed using an OBIS LX 375 nm diode laser with a pulse period of 200 ns and a Hamamatsu H74422–40 photomultiplier tube. For temperature-dependent photoluminescence decay curves, we used an OBIS LX 375 nm laser module operated with a pulse generator with varying pulse widths and repetition rates. The temperature-dependent measurements on NCs down to 4 K were carried out with an Oxford Instruments liquid-He cryostat and a homemade liquid quartz cell to contain the NCs in solution. The low-temperature measurements on MCs were performed with an Oxford Instruments coldfinger liquid-He cryostat.

## Results and Discussion

**Figure 1:**
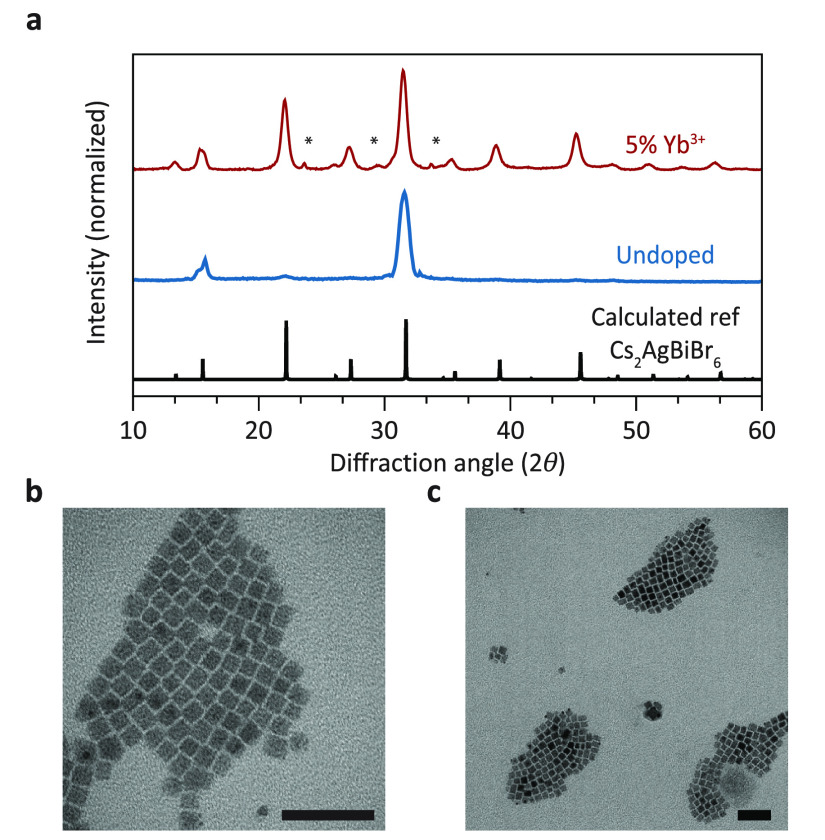
XRD patterns and TEM photographs of the undoped and Yb-doped Cs_2_AgBiBr_6_NCs. (a) XRD patterns show that the doped and undoped NCs crystallize in the cubic phase. The asterisks indicate a small impurity phase, which could be attributed to a ternary Cs–Bi–Br impurity.^26^ The reference pattern has ICSD collection code 239874. TEM images of (b) Cs_2_AgBiBr_6_ NCs and (c) Cs_2_AgBiBr_6_: 0.23% Yb^3+^ NCs. The scale bar is 50 nm in both images.

First, we present and discuss the general structural and optical characteristics of undoped Cs_2_AgBiBr_6_ and Cs_2_AgBiBr_6_:Yb^3+^ NCs that were synthesized using a previously reported hot injection method.^26^ The XRD patterns (Figure 1a) demonstrate that both the doped and undoped NCs adopt a cubic structure with the *Fm*3̅*m* space group, with the (200) and (400) lattice plane reflections being the most prominent. Reflections from other lattice planes are weaker than expected based on the reference pattern, likely due to the preferential ordering of the NCs on a low-background Si wafer. The weaker reflections are also present (Supporting Information, S1) and show that the Cs_2_AgBiBr_6_ NCs with the elpasolite structure have formed. TEM images show that the doped and undoped NCs are monodisperse in size and cubic in shape (Figure 1b,c). These properties, and also the size with an average edge length of 9 ± 0.4 nm, are in good agreement with those of NCs in earlier reports.^20,26^ ICP-OES measurements were conducted to verify the incorporation of Yb^3+^ into the host lattice, which resulted in a 0.23% Yb^3+^ doping concentration relative to that of Bi^3+^ ([Yb^3+^] = [Yb^3+^]/([Yb^3+^]+[Bi^3+^])). The fraction of Yb^3+^ incorporated is much lower than the 5% Yb-to-Bi feeding ratio. The challenge in incorporating lanthanides is consistent with that found in prior studies. Incorporation is especially difficult in NCs as impurities can be more easily removed or prevented from incorporation by staying in solution. The harder Lewis acid nature of Yb^3+^ makes it bind more strongly to oleic acid than the softer Lewis acid Bi^3+^.^28^ Because of the higher energy needed to dissociate Yb^3+^ from the ligands, it is not unexpected that the fraction of Yb^3+^ incorporated into the Cs_2_AgBiBr_6_ NCs is well below the concentration in the reaction mixture.

**Figure 2:**
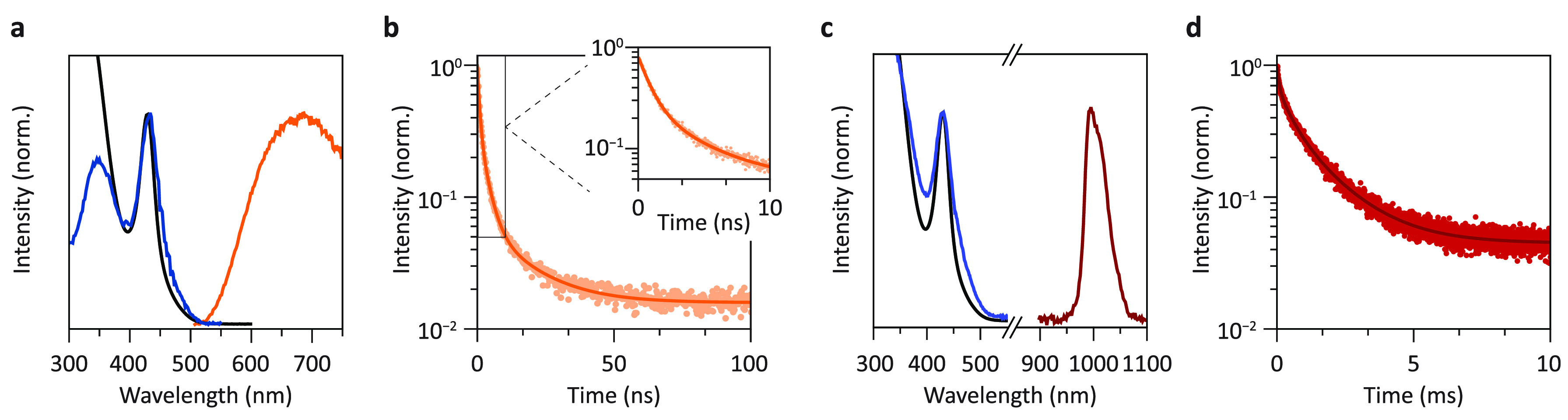
Optical properties of undoped Cs_2_AgBiBr_6_NCs and Cs_2_AgBiBr_6_: 0.23% Yb^3+^NCs at room temperature. (a) Absorption (black line), emission (orange), and excitation (blue) spectra of the undoped Cs_2_AgBiBr_6_ NCs, with λ_exc_ = 420 nm and λ_em_ = 650 nm. (b) Photoluminescence decay curve of the host emission, excited at 374 nm and recorded at 660 nm. The inset shows the fast initial decay component. The data is fitted with an exponential function containing three exponents, from which the weighted average lifetime is calculated with τ_ave_ = Σ_i_*A*_i_ τ_i_/Σ_i_*A*_i_. (c) Absorption and near-IR emission and excitation spectra of the Cs_2_AgBiBr_6_:Yb^3+^ NCs, with λ_ex_ = 350 nm and λ_em_ = 1000 nm. The host emission is still observable but is not shown in the graph. (d) Photoluminescence decay curve of the Yb^3+2^F_5/2_ → ^2^F_7/2_ emission (λ_exc_ = 374 nm and λ_em_ = 1000 nm), fitted with two exponents (dark brown).

Figure 2a shows the room temperature absorption, emission, and excitation spectra of the undoped Cs_2_AgBiBr_6_ NCs. The absorption spectrum reveals a peak at 430 nm that has a gradually increasing and weak absorption onset starting around 520 nm. The strong excitonic peak and weak absorption onset are typically associated with a direct and indirect band gap transition, respectively.^29^ See Supporting Information Section S2 for a Tauc analysis of the absorption spectrum. At shorter wavelengths, the absorption drops beyond the first excitonic transition and then significantly increases at wavelengths shorter than 380 nm. Upon excitation in the exciton peak at 420 nm, weak, broadband emission centered around 690 nm is observed, in agreement with earlier work.^30^ The excitation spectrum of the red emission (λ_em_ = 660 nm) recorded from 300 to 600 nm shows that the excitation and absorption spectra overlap until 350 nm, in good agreement with an indirect and direct excitonic transition. At shorter wavelengths, the excitation intensity is lower in comparison to the absorption spectrum. Such differences between absorbance and excitation intensity are sometimes attributed to physical properties of luminescent materials, which has also been reported for Cs_2_AgBiBr_6_ and has, for example, been explained by the neutral character of the exciton state, which would less effectively populate the emissive state.^22,24,29^ Instead, we attribute this to artifacts related to absorption saturation and/or competing absorption in the ultraviolet. Because the emission of Cs_2_AgBiBr_6_ NCs is weak at room temperature, a high NC concentration is often used to achieve a good signal-to-noise ratio. For high concentrations, strong absorption effects can cause distortions of the excitation spectra by complete absorption of the excitation light in a thin layer of sample. Deeper penetration of the excitation light at wavelengths with less absorption can thus lead to an overall stronger detected emission signal. It is not unusual to even observe a dip at the peak wavelength for the strongest absorption due to this effect as stronger absorption leads to less detected emission. To demonstrate the effect of concentration on experimentally observed excitation spectra, we conducted excitation measurements on a dilution series (for details, see Supporting Information Section S3). The series reveals a redshift of the absorption maximum for higher concentrations and the presence of an excitation dip around the absorption maximum. However, for the strongest dilution, the excitation spectrum closely resembles the absorption spectrum, showing that the change in the shape of the excitation spectrum is not caused by the physical properties (such as a variation in the efficiency of trapped exciton generation, depending on the excitation energy) of the luminescent NCs and that the true excitation spectrum closely follows the absorption spectrum, as expected and in line with earlier observations, including those for the analogous system CsPbCl_3_:Yb^3+^.^31^

Continuing our analysis, luminescence decay curves of the host emission were recorded at room temperature and show a fast nanosecond decay that is best fitted with three exponents (Figure 2b). Based on this fit, the calculated average decay time (τ_ave_ = Σ_i_*A*_i_ τ_i_/Σ_i_*A*_i_) is 2 ns. The multi-exponential decay, in addition to the presence of a fast sub-ns component (inset Figure 2b), is explained by a combination of thermal quenching and quenching caused by surface defects in Cs_2_AgBiBr_6_ NCs.^26^ The radiative decay time for the STE emission is expected to be in the μs range (and is indeed observed at low temperatures^32^), but a combination of thermal quenching and surface quenching causes the luminescence decay curves to be nonexponential with ns components. The origin of the strongly red-shifted photoluminescence in Cs_2_AgBiBr_6_ NCs is often ascribed to the formation of STEs^33,34^ or color centers^29^ after photoexcitation. Upon direct bandgap photoexcitation of an electron from the valence band (VB) to the conduction band (CB), within ps, a trapped exciton state is formed, giving rise to broadband red emission.^35^ During very short periods of time, weak direct bandgap emission in the blue has also been observed, which decays on a ps time scale, giving evidence for the fast relaxation from the direct bandgap exciton state.^33,34^ One conceivable scenario for the rapid capture of charge carriers involves the localization of a VB hole on a [AgBr_6_]^5–^ cluster, leading to lattice relaxation.^35^ The propensity of Ag^+^ to act as hole acceptors has been observed in other semiconductor systems like CdSe doped with Ag^+^.^36^ A STE forms if the trapped hole binds a photoexcited electron. Radiative recombination from this STE state to the ground state is characterized by a large lattice relaxation and offset between the ground and excited-state parabola. As a result, the emission spectrum consists of a broad band with a large Stokes shift. Because of the local differences in coordination of the self-trapped state, decay curves are typically multi-exponential.^25^ As a result of the large Stokes shift, semiconductor elpasolites often display thermal quenching around or below room temperature due to thermally assisted crossover from the excited state to ground-state parabola.^32,37^

Next, we focus on the optical properties of the Yb-doped Cs_2_AgBiBr_6_ NCs and compare them with that of the undoped NCs. There is no variation in the absorption and host emission spectra with respect to the undoped NCs (Figure 2c–Supporting Information Section S4). Due to the low efficiency of the host emission at room temperature, it is difficult to estimate the effect of Yb^3+^ incorporation on the intensity of the host PL. In the near-IR emission spectrum, a sharp peak is observed around 1000 nm for the Yb^3+^-doped sample, which we assign to the ^2^F_5/2_ → ^2^F_7/2_ transition of Yb^3+^. Upon recording an excitation spectrum of the NIR emission band, the spectrum very closely matches the host absorption spectrum, and this proves that the Yb-emission is excited through the Cs_2_AgBiBr_6_ host and that ET from the host to Yb^3+^ occurs. Time-resolved measurements of the Yb emission show that it has a multi-exponential decay that can be fitted with a biexponential decay function (Figure 2d). The weighted average decay time is 1.5 ms. A ms lifetime is typical for parity-forbidden f–f transitions of lanthanides. The lifetime is, however, notably faster than the radiative lifetime of Yb^3+^ in bulk elpasolites (6.3 ms in Cs_2_NaYF_6_: 10% Yb^3+^ and 2.7 ms in Cs_2_AgInCl_6_ MCs^27,38^) but comparable to previous work on Yb-doped Cs_2_AgBiBr_6_ NCs.^20^ We explain the multi-exponential and relatively fast decay by quenching of the emission for near-surface Yb^3+^ ions. An additional washing step with acetonitrile was performed to verify whether there is surface-absorbed Yb^3+^ that contributes to the faster decay, but extra washing did not lead to a significant decrease in Yb emission. The Yb^3+^ luminescence is therefore assigned to the Yb^3+^ incorporated in the Cs_2_AgBiBr_6_ NCs. High-energy (∼3000 cm^–1^) C–H vibrations of the OA capping and toluene solvent molecules can quench the Yb^3+^ emission for Yb^3+^ ions close to the surface by multiphonon relaxation as 3 or 4 vibrations can bridge the energy gap between the ^2^F_5/2_ excited state and the ^2^F_7/2_ ground state of Yb^3+^. The distribution of Yb^3+^ through the NCs gives rise to Yb^3+^ ions with longer lifetimes in the center and shorter lifetimes for Yb^3+^ near the surface and, thus, explains the multi-exponential decay.

**Figure 3:**
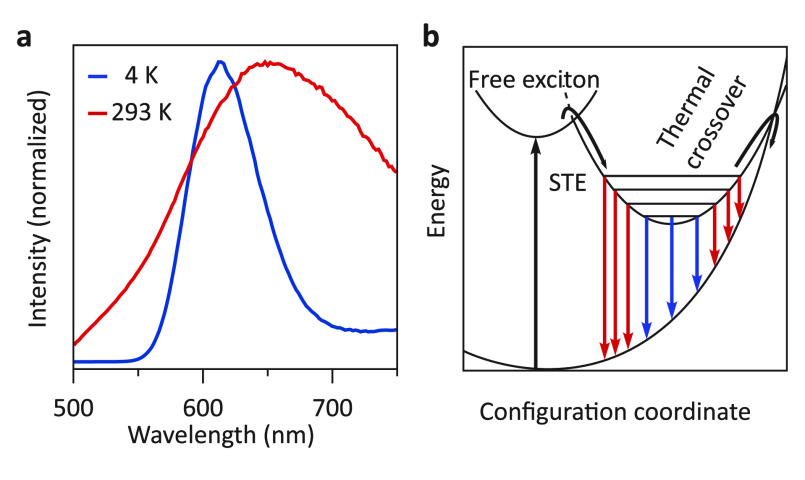
STE emission at room temperature and cryogenic temperature. (a) Emission spectra of Cs_2_AgBiBr_6_ NCs at 4 K and room temperature excited with 374 nm light. (b) Configuration coordinate diagram showing thermal crossover quenching of the STE state and emission from the different vibrational levels. The colors of the emission arrows correspond to the spectra in (a).

Before comparing the temperature-dependent optical properties of the doped and undoped Cs_2_AgBiBr_6_ NCs, we first discuss the temperature-dependent optical properties of the undoped Cs_2_AgBiBr_6_ NCs. Figure 3a shows the host emission at room temperature (RT) and at 4 K. As the temperature increases from 4 K to RT, the emission peak redshifts by ∼50 meV, which is typically observed for semiconductor materials and described empirically by the Varshni equation.^39^ The redshift is accompanied by pronounced peak broadening, exhibiting a full width at half-maximum (fwhm) of 200 meV at 4 K and 600 meV at RT. With increasing temperature, we observe a large decrease in the emission intensity caused by thermal quenching. A similar but smaller peak broadening (fwhm of 125 meV at 4 K and 175 meV at RT) is observed for the absorption spectrum, indicating the much stronger coupling to the lattice of the STE state.^35^ At 4 K, only the lowest vibrational levels of the STE parabola are occupied, which has two effects (Figure 3b): first, crossover to the ground-state parabola does not occur as the wave functions of the lowest vibrational levels of the excited state have a close-to-zero overlap with high vibrational levels of the ground state. Second, emission occurs only from the lowest vibrational states of the excited state, resulting in a relatively narrow emission peak, indicated by the blue arrows. The absence of a zero-phonon line and vibronic fine structure at 4K can be attributed to and is typical for optical transitions with very large electron–phonon coupling.^40,41^ At higher temperatures, higher vibrational states are populated and emission occurs from these levels, leading to a broadening of the emission peak and, ultimately, thermally activated quenching. It is noteworthy that at 4 K, we do not observe free or bound exciton recombination close to the absorption band onset, which indicates that the energy barrier between the free exciton state and the STE state is very small. This finding is in line with the recently observed barrier-free and fast (<ps) charge carrier localization in vapor-deposited thin film Cs_2_AgBiBr_6_.^35^

As discussed above, several mechanisms have been proposed for the ET from the Cs_2_AgBiBr_6_ host to Yb^3+^ dopants, including transfer from the direct bandgap exciton state or the STE state or electron trapping by Yb^3+^ (forming Yb^2+^), followed by release of the electron to the CB, leaving Yb^3+^ in the excited ^2^F_5/2_ state.^24^ In the discussion of our results, we focus on two plausible mechanisms for the ET process: (1) after photoexcitation, the CB electron is trapped by Yb^3+^ ion, reducing it to Yb^2+^ ion. Alternatively, the electron may be trapped near the Yb^3+^ site due to a local lattice deformation. Subsequent charge recombination with a hole results in Yb^3+^ remaining in the excited state.^20,34^ Note that in the mechanism suggested in ref [^24^], it is unclear how the release of the Yb^2+^ trapped electron back to the CB would leave Yb^3+^ in the excited state. (2) Photoexcitation creates an exciton that rapidly relaxes to the red-emitting STE state.^42^ Subsequently, the localized STE state transfers its energy to a nearby Yb^3+^ ion. Both possibilities have been suggested in the literature for lanthanide-doped elpasolite (nano)crystals. A third mechanism where transfer occurs from the unrelaxed direct exciton state seems unlikely as ps relaxation occurs and there is no resonant excited state of Yb^3+^ to which ET can occur given the fact that the ^2^F_5/2_ state is the only intra 4f^13^ excited state of Yb^3+^.

**Figure 4:**
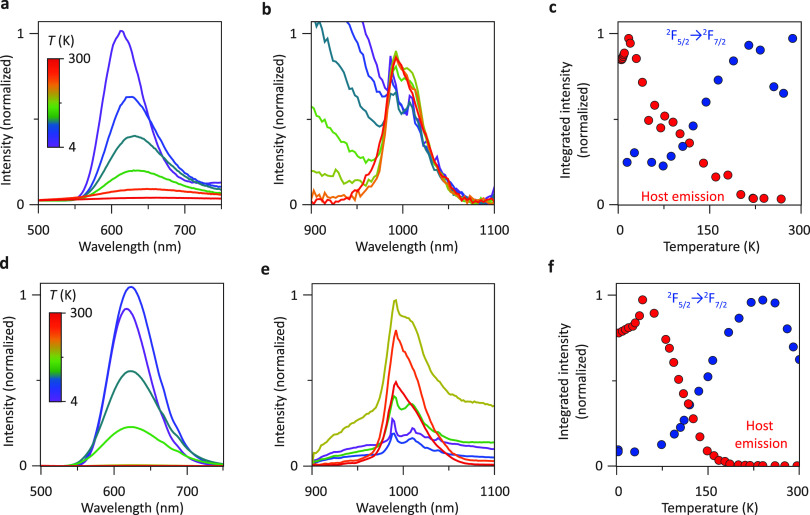
Temperature-dependent emission spectra of Yb^3+^-doped Cs_2_AgBiBr_6_NCs (a–c) and MCs (d–e). (a) Temperature-dependent emission spectra of the host emission intensity measured between 4 and 267 K. Upon increasing the temperature, the emission band redshifts, becomes broader, and gets increasingly quenched. (b) Temperature-dependent Yb^3+^ emission measurements from 7 to 300 K. Note that the red tail of the host emission band starts overlapping with the Yb emission at lower temperatures. (c) Temperature dependence of the integrated host emission intensity and Yb^3+^ emission intensity. Temperature-dependent (d) visible and (e) NIR emission spectra of Cs_2_AgBiBr_6_: Yb^3+^ MCs. (f) Integrated intensity of the visible and NIR emission of Cs_2_AgBiBr_6_: Yb^3+^ MCs as a function of temperature. The excitation wavelength for all experiments was 374 nm.

To distinguish between the different mechanisms, temperature-dependent luminescence and time-resolved spectroscopy experiments were conducted. Figure 4a–c presents the temperature-dependent emission spectra of the Yb^3+^-doped Cs_2_AgBiBr_6_ NCs. The temperature dependence of the host emission in Figure 4a shows the same trend with increasing temperature as the undoped sample (Figure 3a–Supporting Information Section S5) and is almost completely quenched at room temperature. Interestingly, the Yb^3+^ emission intensity shown in Figure 4b increases when the temperature increases. Also, because of line narrowing at low temperatures, multiple sharp emission lines become visible in the low-temperature emission spectra around 1000 nm due to the crystal field splitting of the ^2^F_5/2_ and ^2^F_7/2_ levels. To accurately determine the intensity of the Yb emission at low temperatures, the background of the host emission band has to be subtracted as the host emission band becomes more intense with decreasing temperatures and the tail overlaps with the Yb^3+^ line emission. The procedure for background correction is described in detail in Supporting Information Section S6. The temperature dependence of the integrated intensities of both host and Yb emissions is summarized in Figure 4c, where the normalized and background-corrected emission integrals of both the host and Yb emission bands are plotted. The red host emission (originating from the STE state) increases in intensity from 4 K up to 20 K, after which it strongly quenches due to thermally activated nonradiative processes. The intensity of the Yb band increases 4-fold, going from 50 to 250 K, after which the intensity drops by about 30% up to 300 K. These results strongly suggest that the STE state does not act as a sensitizer in the ET process as in mechanism (2). If ET would occur via the STE state, the Yb emission intensity is expected to experience similar thermal quenching. Instead, the opposite is observed.

Because of the presence of surface quenching sites at NC surfaces, it is instructive to compare the properties of nanocrystalline Cs_2_AgBiBr_6_:Yb^3+^ with that of its microcrystalline analogue (XRD in Supporting Information Section S7). Therefore, we synthesized and measured Yb-doped Cs_2_AgBiBr_6_ MCs with a 5% Yb feed concentration (with respect to Bi^3+^), and we measured its temperature-dependent emission spectra (Figure 4d–f). The exciton Bohr radius in perovskites and elpasolites is typically smaller than 5 nm, and the effect of quantum confinement on the host emission is very limited for ∼10 nm NCs.^43,44^ Indeed, the similar host emission band energy at 4K for the doped NCs and MCs (Figure 4d) indicates no role of confinement effects. In that situation, the sensitization mechanism in NCs and MCs is expected to be the same. The temperature-dependent red host emission and Yb^3+^ NIR emission presented in Figure 4e show a trend and features similar to those in the NCs. After an initial increase at low temperatures, the host emission strongly drops between 50 and 200 K, while the Yb^3+^ NIR emission increases by a factor of 13 in that same temperature range. For the MCs underneath the Yb-emission band, a broad emission band is present that also varies in intensity with temperature (details in Supporting Information Section S8). The emission intensity related to Yb^3+^ was determined by a background correction procedure similar to that discussed for Figure 4b. The origin of the broad emission band is unclear, and this NIR emission is not observed in the Cs_2_AgBiBr_6_ NCs. Cu-doped Cs_2_AgBiBr_6_ single crystals did show a similar broad NIR emission peak with the same temperature dependence, suggesting that Cu-impurities may be the origin.^45^ In Figure 4f, we again compare the integrated emission intensities of both the MC host and Yb-related emission as a function of temperature and observe characteristics very similar to those in the NCs. There is an increase in emission intensity above 4 K in both the Cs_2_AgBiBr_6_ NCs (to 20 K) and MCs (to 40 K) followed by strong thermal quenching, which we discuss below together with the time-resolved measurements below. In the MCs, the PL intensity of the Yb emission shows a similar temperature dependence to that in the NCs. These results highlight the fact that the Yb sensitization mechanism is the same in NCs and MCs.

**Figure 5:**
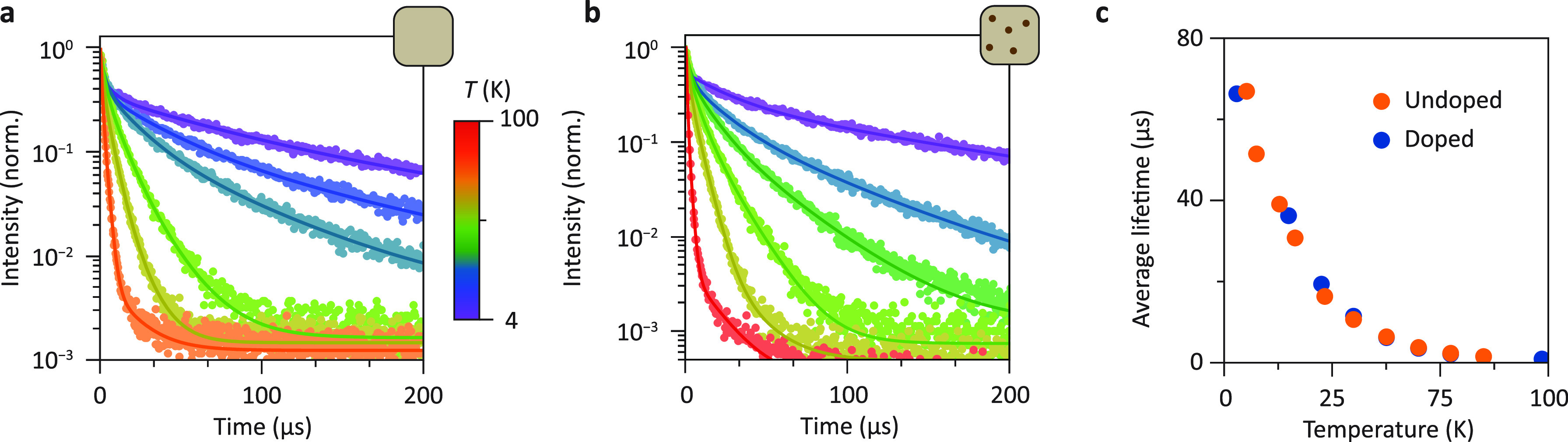
Temperature-dependent photoluminescence decay measurements of the host emission of doped and undoped Cs_2_AgBiBr_6_NCs. (a) Photoluminescence decay measurements of undoped Cs_2_AgBiBr_6_ NCs between 6 and 80 K. (b) Host emission lifetime of Yb-doped Cs_2_AgBiBr_6_ NCs from 4 to 100 K. The excitation wavelength for both measurements is 374 nm, and the emission wavelength is shifted with the emission peak maximum. In order to capture the increasingly fast decay with increasing temperature, various laser repetition rates between 400 Hz and 10 kHz were used to record the decay curves. (c) Weighted average of the decay lifetime as a function of temperature for Yb-doped and undoped Cs_2_AgBiBr_6_.

To further investigate the ET pathway from the host to Yb^3+^, we performed variable temperature PL lifetime measurements from 4 to 100 K on both doped and undoped NCs (Figure 5a–b). Because of the large changes in decay time, the decay curves are shown up to 200 μs, even though at 4 K, this does not capture the complete luminescence decay behavior. Full decay curves and further details about the fitting procedure and the presence of a fast component can be found in Supporting Information Section S9. Similar to the room temperature decay curves discussed above, the luminescence decay of the undoped NCs is multi-exponential, revealing the presence of multiple decay pathways.^46^ In order to determine decay times, a three-exponential fit procedure was used to calculate the weighted average lifetime. The resulting average lifetimes are shown in Figure 5c. To obtain insights into the role of direct ET from the STE to Yb^3+^, the host emission lifetimes of the doped and undoped NCs are compared. We observe no difference between the average lifetimes of the host emissions for the doped and undoped Cs_2_AgBiBr_6_ NCs, which provides further evidence that the ET mechanism does not involve the STE state as an intermediary. If ET would occur from the STE state, a shorter emission decay time would be expected for the STE emission in the Yb-doped NCs as ET is an additional decay pathway.

The rapid drop in the STE emission lifetime between 4 and ∼50 K from 65 to 5 μs cannot be explained by thermal quenching as, in this temperature regime, there is even an increase in emission intensity. A plausible explanation is the spin-forbidden nature of emission from the lowest-energy, high-spin STE dark state. Upon raising the temperature, the thermal population of the low-spin bright state allows the emission, and this gives rise to faster radiative decay. This is generally observed for (trapped) exciton emission.^47,48^ The strong rise in the radiative decay rate can also explain the increase in emission intensity in the low-temperature regime. If there are nonradiative decay pathways with no or weak temperature dependence, at elevated temperatures, faster radiative rates will favor radiative decay over nonradiative decay and the emission intensity increases. Above 50 K, thermal quenching of the STE emission starts, and a rapid decrease in emission intensity and further decrease in the emission decay time to 2 ns at room temperature is observed. The thermal quenching of STE emission from Cs_2_AgBiBr_6_ has been observed before, and the present results are in line with previous observations.^32^

**Figure 6:**
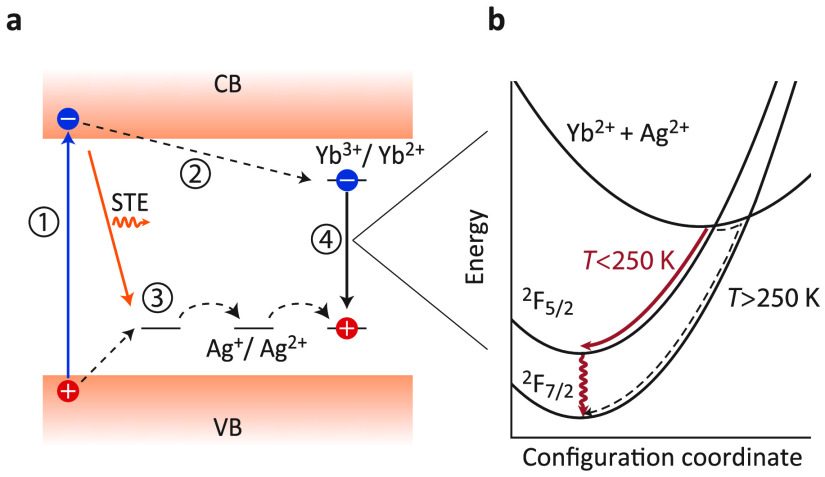
Schematic representation of the emission mechanism of Yb^3+^-doped Cs_2_AgBiBr_6_NCs and MCs. (a) Band diagram of Cs_2_AgBiBr_6_ with the all the transitions leading to Yb^3+^ emission marked with arrows. Formation of an STE state is in competition with trapping on Yb^3+^. Red STE luminescence is denoted by the orange arrow. (**1**) Indicates the absorption of blue light leading to the generation of charge carriers. (**2**) CB electron traps on Yb^3+^ and their reduction to Yb^2+^. (**3**) The VB hole can localize on a [AgBr_6_]^5–^ cluster effectively oxidizing Ag^+^ into Ag^2+^. The trapped hole can migrate through the lattice through a temperature-activated process. (**4**) Recombination of a trapped hole and electron happens through the Yb^2+^ + Ag^2+^ → Yb^3+^ + Ag^+^ CT. (b) Configuration coordinate diagram of Yb^3+^ on a Bi^3+^ site. The upper parabola represents a CT state where Yb^3+^ is reduced to the oxidation state (II), accompanied by a hole that localizes on an Ag^+^ site. The CT step between Yb^2+^ and Ag^2+^ leaves the Yb^3+^ in the ^2^F_5/2_ excited state, after which relaxation to the ^2^F_7/2_ ground state takes place. With increasing temperature (roughly above 250 K), there is a diminishing probability of forming Yb^3+^ in the excited ^2^F_5/2_ state after the CT step.

Based on the temperature-dependent luminescence properties and decay dynamics for undoped and Yb^3+^-doped Cs_2_AgBiBr_6_ NCs and MCs, we can construct a mechanism for the host-mediated photoexcitation of Yb^3+^ in Cs_2_AgBiBr_6_ NCs and MCs (Figure 6a). Initially, after direct bandgap absorption of a photon, a VB electron is promoted to the CB, creating an electron–hole pair. The hole subsequently localizes on a [AgBr_6_]^5–^ cluster on a ps time scale,^35^ while the electron remains delocalized in the NC (or MC). The subsequent trapping of the electron can take place in multiple ways, but a recent photoconductivity study showed that almost all electrons do localize on trap sites on a sub-ns time scale.^49^ Upon doping Cs_2_AgBiBr_6_ NCs and MCs with Yb^3+^, a competing pathway for electrons is trapping by Yb^3+^, forming Yb^2+^, or an Yb^3+^ impurity trapped electron state. Recombination with a trapped hole results in Yb^3+^ in the excited ^2^F_5/2_ state, followed by the characteristic sharp line emission from Yb^3+^ around 1000 nm corresponding to the ^2^F_5/2_ → ^2^F_7/2_ f–f transition. The negative thermal quenching behavior of the Yb^3+^ emission provides support for this mechanism. It is well-known that as the temperature increases, the mobility of trapped carriers in Cs_2_AgBiBr_6_ (and, for example, also AgCl) increases.^35,50,51^ The higher hole mobility increases the probability for recombination at a Yb^2+^ site, thereby leaving the Yb^3+^ ion in the excited state and causing an increase in the characteristic ^2^F_5/2_→ ^2^F_7/2_ emission intensity (Figure 6b). A similar mechanism has been proposed and experimentally validated for Yb^3+^ emission in InP thin films.^52,53^ In this model, the subsequent decrease in the Yb^3+^ emission intensity at even higher temperatures, above 250 K, can be explained by thermally activated relaxation from the ^2^F_5/2_ state to the ^2^F_7/2_ ground state via the CT state.

Finally, it is interesting to consider the nature of the trapped electron state, specifically whether Yb^2+^ is formed or whether electron trapping occurs as a result of a lattice distortion near the Yb^3+^ impurity. A similar discussion is valid for Yb^3+^-doped perovskite halides where the ET mechanism to Yb^3+^ is also the topic of debate.^6,31^ The position of the Yb^2+^ ground state relative to the CB minimum is relevant. Often, the energetic position of Yb^2+^ relative to the VB is estimated from the energy of the CT absorption band.^54^ Indeed, the CT absorption corresponds to the excitation of a VB electron to Yb^3+^. The maximum of the CT absorption band for Yb^3+^ in bromides is typically 3–3.5 eV,^54−56^ which would suggest that the Yb^2+^ ground state cannot be situated in the forbidden gap in Cs_2_AgBiBr_6_ as the bandgap energy is only 2.4 eV. However, one has to realize that the CT absorption band maximum corresponds to a transition to a very high vibrational level in the CT excited state. CT transitions are characterized by strong lattice relaxation giving rise to broad emission and excitation bands and large Stokes shifts, typically around 2 eV for Yb^3+^ CT transitions.^57^ The position of the Yb^2+^ ground state in the energy band diagram corresponds to the relaxed state and not the energy of the CT absorption band maximum. Stabilization of the Yb^2+^ CT state as the system relaxes to the new equilibrium distances has to be taken into account (but is often forgotten in band diagram pictures that do not allow for depicting lattice relaxation). The lattice relaxation in the excited CT state is about half the Stokes shift, around 1 eV for Yb^2+/3+^, and thus, the position of the Yb^2+^ trapped electron level may very well be located in the forbidden gap of Cs_2_AgBiBr_6_, just below the CB minimum. Further research is needed to pinpoint the nature of the trapped electron state.

## Conclusions

To conclude, we have investigated the optical properties of undoped and Yb^3+^-doped Cs_2_AgBiBr_6_ NCs and MCs at variable temperatures down to 4 K. Both broadband trapped exciton emission around 690 nm and NIR Yb^3+^ line emission around 1000 nm are observed. The Yb^3+^ emission can be successfully excited through Cs_2_AgBiBr_6_ host absorption, as confirmed by excitation spectroscopy. Surprisingly, the Yb emission intensity as a function of temperature shows a negative thermal quenching in both Cs_2_AgBiBr_6_ NCs and MCs, while the red trapped exciton emission shows strong thermal quenching above 50 K. Temperature-dependent emission and time-resolved spectroscopy confirm that the STE emission is not strongly affected by the incorporation of Yb. These results can be explained by a host-to-Yb ET mechanism in Cs_2_AgBiBr_6_ that does not take place via the red-emitting trapped exciton state but by electron trapping on Yb^3+^ in competition with forming a STE state. The subsequent recombination of Yb^2+^ with a trapped hole results in Yb^3+^ in the excited ^2^F_5/2_ state and characteristic Yb^3+^ sharp line emission around 1000 nm due to the ^2^F_5/2_ → ^2^F_7/2_ 4f–4f transition. Thermally activated hole mobility explains the negative thermal quenching of the Yb^3+^ emission. Based on these results, we provide evidence for host-to-Yb ET in Cs_2_AgBiBr_6_ by charge carrier trapping on Yb^3+^ and not through the STE state.

## Supporting Information Available

XRD Cs_2_AgBiBr_6_ NCs and MCs, Tauc plot analysis, influence of NC concentration on excitation spectra, comparison of doped/undoped Cs_2_AgBiBr_6_ NCs, NIR background correction for NCs and MCs, and all temperature-dependent decay curves (PDF)
