# Supplementary material for: Shedding Light on Host-to-Yb3+ Energy Transfer in Cs2AgBiBr6:Yb3+ (nano)crystals
Source: Chem Mater. 2024 Mar 4;36(6):2857–66. doi: 10.1021/acs.chemmater.3c03201 (PMC10976640; doi:10.1021/acs.chemmater.3c03201)
Supplement: Supplementary file 1 — cm3c03201_si_001.pdf [file cm3c03201_si_001.pdf]

Supporting Information

Shedding light on host-to-Yb^{3+} energy transfer in $Cs_2AgBiBr_6$:Yb^{3+} (nano)crystals

Jur W. de Wit[1], *Lars L. Sonneveld*[1] *and Andries Meijerink*[1*]

[1]*Debye Institute for Nanomaterials Science, Utrecht University, Princetonplein 1, 3584 CC Utrecht, The Netherlands,*

[*] Corresponding author. E-mail: a.meijerink@uu.nl

Contents

Section S1. XRD patterns of doped and undoped $Cs_2AgBiBr_6$ NCs

Section S2. Tauc plot analysis of $Cs_2AgBiBr_6$ NCs

Section S3. The effect of NC concentration on $Cs_2AgBiBr_6$ excitation spectra

Section S4. Host emission spectrum of undoped and doped $Cs_2AgBiBr_6$ NCs

Section S5. Temperature-dependent emission spectrum of undoped and doped $Cs_2AgBiBr_6$ NCs

Section S6. Background correction for temperature dependent NIR emission spectra of $Cs_2AgBiBr_6$:Yb^{3+} NCs

Section S7. XRD patterns of $Cs_2AgBiBr_6$ and $Cs_2AgBiBr_6$:Yb^{3+} MCs

Section S8. Background correction for temperature-dependent NIR emission spectra of $Cs_2AgBiBr_6$:Yb^{3+} MCs

Section S9. Temperature-dependent lifetime measurements of doped and undoped $Cs_2AgBiBr_6$ NCs

Section S1. XRD patterns of doped and undoped $Cs_2AgBiBr_6$ NCs

The XRD patterns for the undoped (figure S1a) NCs only reveals all $Cs_2AgBiBr_6$ reflections upon zooming in, in contrast to the XRD pattern recorded for the $Cs_2AgBiBr_6$: 0.23% Yb^{3+} NCs (figure S1b). The difference originates from the change in measurement settings. The pattern of the undoped sample was recorded with a total recording time of 12 min. The XRD pattern of the Yb-doped NCs were recorded with a total integration time of 11 h.

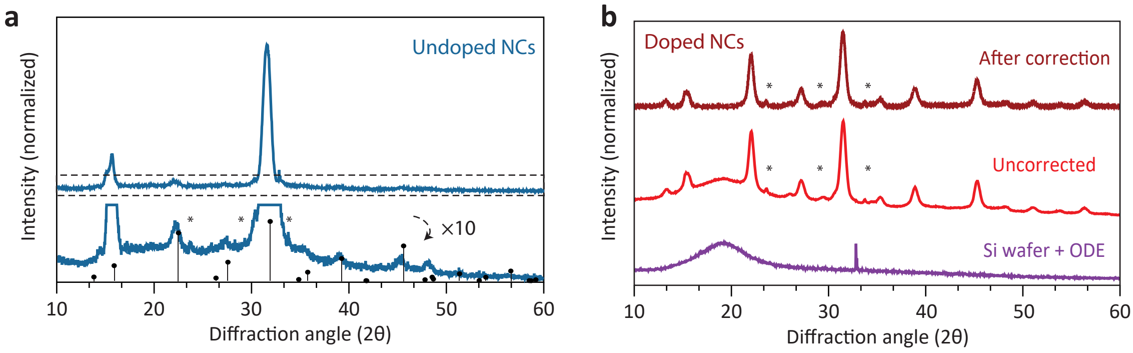

Figure S1 |XRD patterns of the doped and undoped $Cs_2AgBiBr_6$ NCs. XRD patterns for the **a** $Cs_2AgBiBr_6$ and **b** $Cs_2AgBiBr_6$: 0.23% Yb^{3+} NCs. A significant amount of material from one NC synthesis was used for obtaining an XRD measurement with sufficient signal. In order to have a high concentration of NCs, only the standard purification steps were performed, not washing with acetonitrile. This results in a higher concentration of ODE in the solution and gives rise to an additional broad band around 19 degree (see reference pattern from ODE). In the corrected XRD pattern this background is subtracted. The asterisks denote minor impurities in the sample that originate from a ternary Cs-Bi-Br impurity.[1]

Section S2. Tauc plot analysis of $Cs_2AgBiBr_6$ NCs

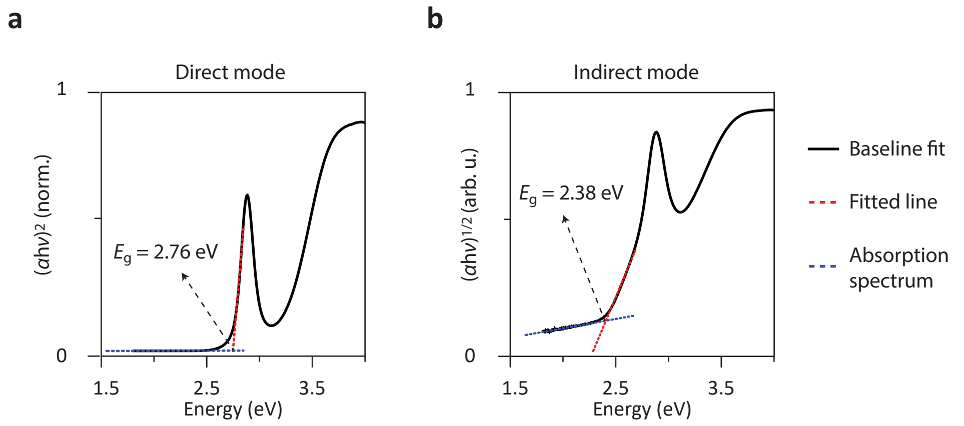

Figure S2 |Tauc plot analysis of the absorption spectrum of undoped $Cs_2AgBiBr_6$ NCs based on ref [2] (**a**) Plot of $(\alpha h\nu)^2$ vs. E to determine the direct bandgap at the crossing point between the baseline (fitted between 1.5 and 2.5 eV) and slope between 2.75 and 2.85 eV. (**b**) Tauc analysis of the indirect bandgap from the plot of $(\alpha h\nu)^{0.5}$ vs. E. Again the crossing point between the background and slope (fitted between 2.4 and 2.68 eV) is determined and gives the indirect bandgap energy.

Section S3. The effect of NC concentration on $Cs_2AgBiBr_6$ excitation spectra

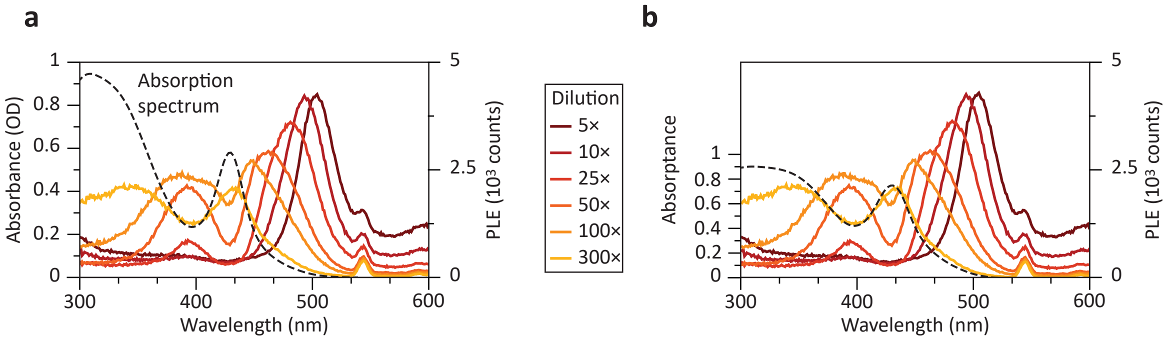

Figure S3 | Absorbance (a) and absorptance (b) plotted with photoluminescence excitation (PLE) spectra for different dilutions of the $Cs_2AgBiBr_6$: 0.23% Yb^{3+} NCs. Both the absorbance ($A = -log(T)$) and the absorptance ($\alpha = 1 - T - R$, where T is the transmittance and R is the reflectance) since the PLE intensity scales with absorptance (linear) instead of absorbance (logaritmic).[3] Differences between absorptance spectra and excitation spectra thus reflect absorption processes that do not give rise to (detected) emission of photons. Excitation spectra for emission at 650 nm were recorded for decreasing concentrations of the NCs in solution, up to a factor 300. The effect of NC concentration on the excitation spectrum can clearly be seen. Only at a dilution of 300× with respect to the concentrated stock solution the excitonic peak of the excitation spectrum aligns with the absorption/absorptance spectrum. The red shift and distortions (e.g. dip at maximum absorption) at higher concentrations are explained by saturation effects and the excitation spectrum for the highest dilution represents the true excitation spectrum. Note that also for this strongly diluted sample at wavelengths < 360 nm the PLE intensity is lower than expected based on the absorptance. One possible explanation is that the high absorptance (>0.8) causes additional artefacts, like discussed in the supporting information of ref 4. It can also because of competing UV absorption by other molecules formed during the reaction which does not lead to 650 nm emission. The peak around 540 nm is a Raman peak. This was checked by shifting the recording emission wavelength. This resulted in a similar shift (in energy) of the peak around 540 nm in the excitation spectrum. The energy difference between the 540 nm peak and the 'emission' wavelength of 650 nm is 3000 cm^{-1}, consistent with C-H stretching vibrations and confirms that this is a Raman peak.

Section S4. Host emission spectrum of undoped and doped $Cs_2AgBiBr_6$ NCs

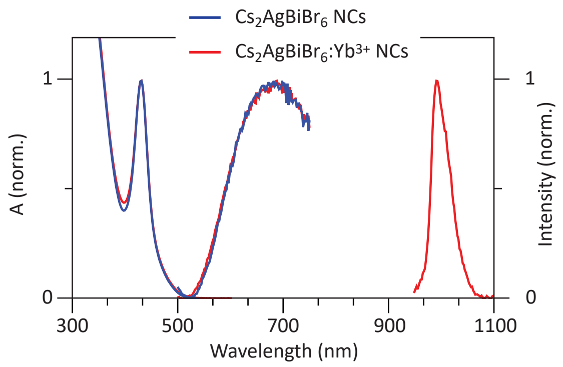

Figure S4 | Normalized room temperature absorption and emission of Yb-doped (red) and undoped (blue) $Cs_2AgBiBr_6$ NCs. Emission spectra were recorded with 430 nm excitation at room temperature.

Section S5. Temperature-dependent emission spectrum of undoped and doped $Cs_2AgBiBr_6$ NCs

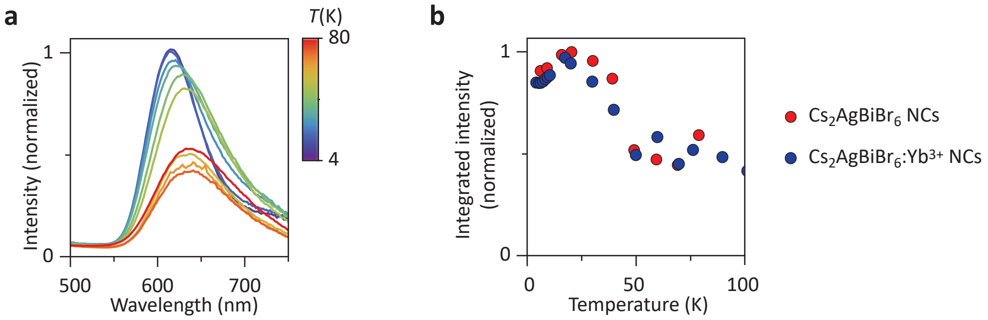

Figure S5 | Temperature-dependent emission of undoped $Cs_2AgBiBr_6$ NCs between 4 and 80 K. (**a**) Emission spectra were recorded with 374 nm excitation. (**b**) Integrated intensity plotted as a function of temperature. The comparison with the emission intensity measured for the $Cs_2AgBiBr_6$:Yb^{3+} NCs shows that the quenching behavior is the same for both materials.

Section S6. Background correction for temperature-dependent NIR emission spectra of $Cs_2AgBiBr_6:Yb^{3+}$ NCs

The NIR emission band of Yb-doped $Cs_2AgBiBr_6$ NCs overlaps with the host emission band at increasingly low temperatures. To be able to evaluate the temperature dependence of the emission intensity of the Yb-ions the underlying emission band is fitted with a polynomial function of the form $p(x) = a + bx + cx^2 + dx^3 + ex^4$, where *a, b, c, d*, and *e* are free parameters to determine the background. The integral for the Yb-emission that is shown in figure 4c, is taken between 950 and 1050 nm after subtraction of this background.

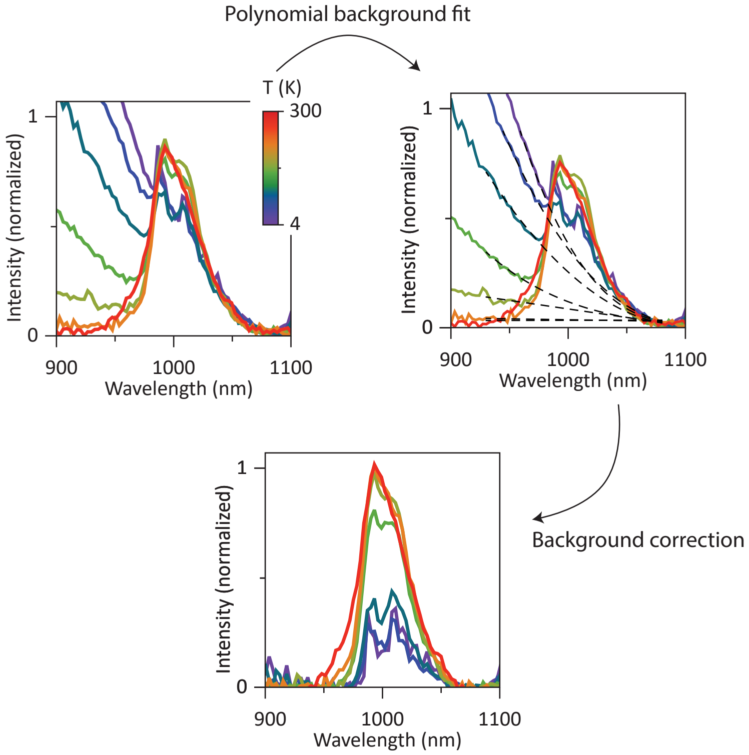

Figure S6 | Polynomial background correction of the T-dependent NIR emission spectra of Yb-doped $Cs_2AgBiBr_6$ NCs. (top left) Original spectra with only the noise of the detector subtracted. (**top right**) Spectra along with the polynomial background fits. The spectral regions to which the polynomial was fitted are 900–940 and 1090–1100 nm. (**Bottom**) Yb^{3+} emission spectra after subtraction of the area underneath the polynomial fit.

Section S7. XRD patterns of Cs2AgBiBr6 and $Cs_2AgBiBr_6{:}Yb^{3+}$ MCs

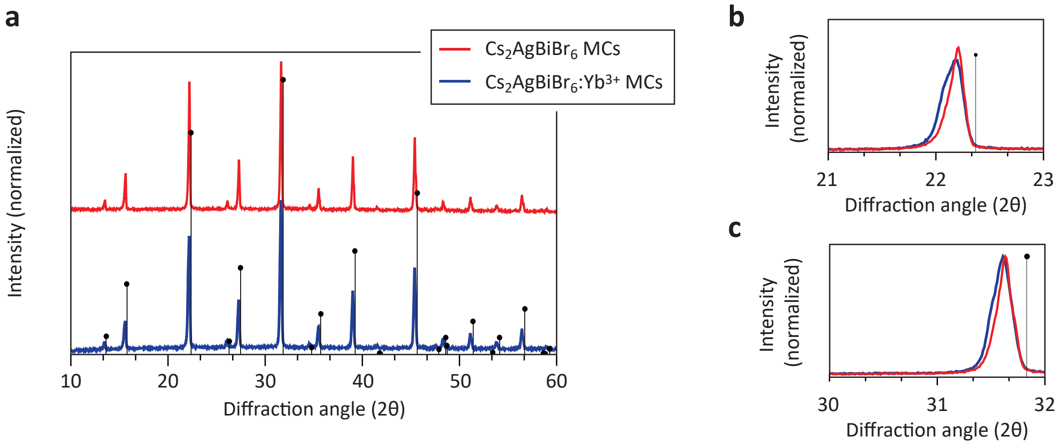

Figure S7 | XRD patterns of $Cs_2AgBiBr_6$ and $Cs_2AgBiBr_6{:}Yb^{3+}$ MCs. (**a**) XRD patterns of doped and undoped $Cs_2AgBiBr_6$. The reference pattern is calculated using VESTA and based on the ICSD collection code 239874. Note that there is a small offset between the peaks of the reference pattern and our experimental data. The offset is probably the result of a small misalignment of the z-height of the measurement stage. (**b**) and (**c**) Zoom-ins on the two main reflections of both doped and undoped $Cs_2AgBiBr_6$ MCs. The peak position of the $Cs_2AgBiBr_6{:}Yb^{3+}$ MCs shows a small shift to smaller angles, which is contrary to what one would expect based on the smaller ionic radius of Yb^{3+} (VI: 0.868) than of Bi^{3+} (VI: 1.03).[5] We therefore conclude that this is a consequence of small differences in z-stage alignment. In the zoom-ins, a small asymmetry is visible in the Yb-doped sample, indicative of increased disorder in the crystal structure.

Section S8. Background correction for T-dependent NIR emission spectra of $Cs_2AgBiBr_6:Yb^{3+}$ MCs

For the NIR emission spectra of the MCs, the background consists of a broad emission band that stretches from roughly 900 to 1500 nm. To determine the temperature dependence of the Yb^{3+} emission a similar procedure as for the NCs was used. The broad band was fitted with a polynomial function of the form $p(x) = a + bx + cx^2 + dx^3 + ex^4$, where *a, b, c, d,* and *e* are free parameters to determine the background. The integral for the Yb-emission that is shown in **figure 6c**, is taken between 950 and 1050 nm after subtraction of this background.

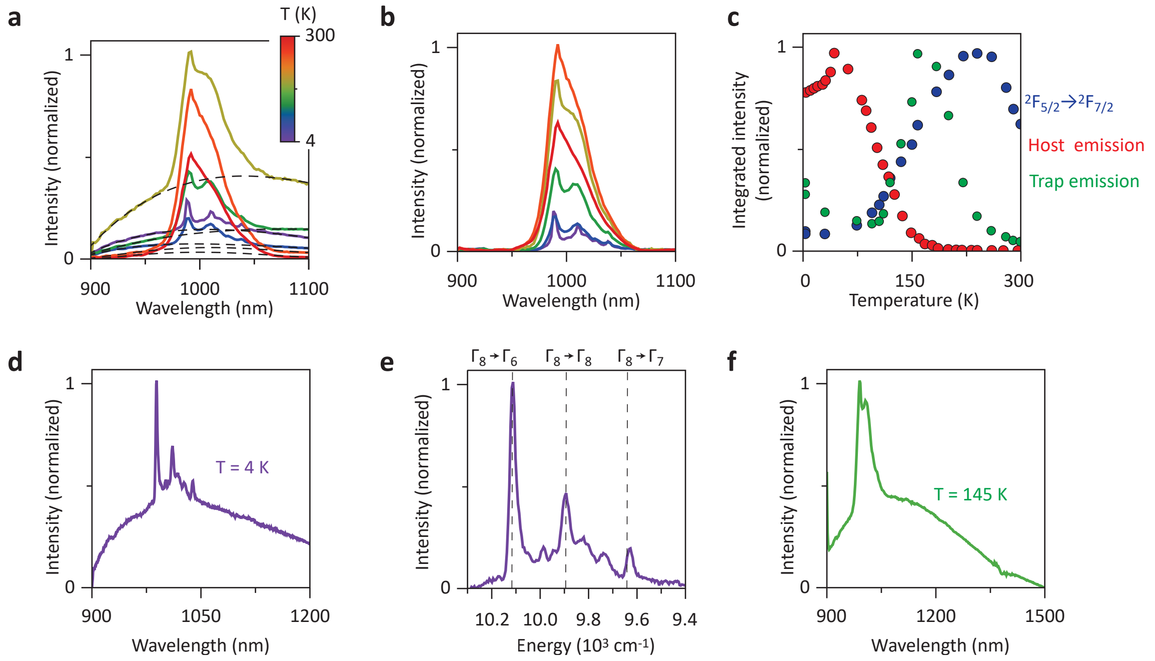

Figure S8 | Temperature-dependent emission spectra of $Cs_2AgBiBr_6:Yb^{3+}$ MCs. (**a**) Emission spectra with only the noise of the detector subtracted and the polynomial fits added. The spectral regions to which the polynomial was fitted are 900–940 and 1090–1100 nm. (**b**) Spectra corrected by subtracting the fits from panel **a**. (**c**) Integrated intensities of the host emission (**figure 4d**, main text), Yb-emission and integrated area underneath the polynomial fits (trap emission). Note that the NIR trap emission intensity peaks around 170 K. (**d**) Higher spectral resolution NIR emission spectrum at 4 K without background correction. (**e**) NIR emission spectrum at 4 K with background correction and peaks assigned to the different crystal field components of Yb^{3+}, the transition energies are obtained from Yb^{3+} in Cs_2NaYCl_6.[6] (**f**) NIR emission spectrum at 4 K without background correction and a wider spectral range (900–1500 nm), indicating that the trap emission band stretches far into the NIR.

Section S9. Temperature-dependent lifetime measurements of doped and undoped $Cs_2AgBiBr_6$ NCs

The temperature-dependent time-resolved emission measurements on both $Cs_2AgBiBr_6$ and $Cs_2AgBiBr_6$: Yb^{3+} are shown in the figures below. As the excitation source an OBIS LX375nm diode laser with a pulse period of 200 ns and its excitation wavelength at 374 nm was used. At the end of a laser pulse, the fall time at the end is specified to be 5 ns. Therefore, analysing decay processes faster than5 ns in these measurements is not feasible. After a decay curve is measured, we fit the data with three exponents and a constant (background) to fully capture the multi-exponential decay. The results of the weighted average are plotted in **figure 5c** of the main text.

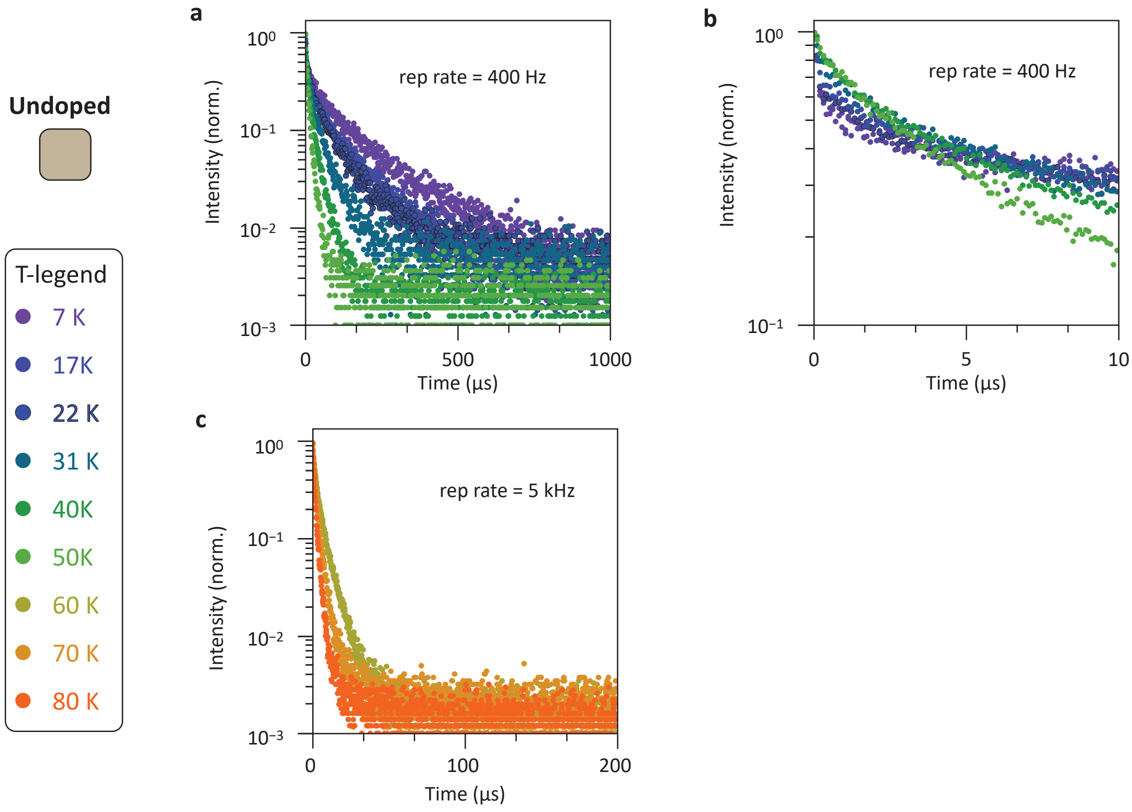

Figure S9 | Temperature-dependent luminescence decay curves of undoped $Cs_2AgBiBr_6$ NCs. (**a**) Decay curves recorded between 4 and 50 K, with a repetition rate of 400Hz for excitation at 374 nm and emission at the maximum of the broad red emission band. Note that on this timescale there is a fast component visible at the beginning of each decay curve. (**b**) Zoom-in on the first 10 µs, revealing a fast initial component present at lower temperature. Previous results in $Cs_2AgBiBr_6$ single crystals showed similar results upon lowering the temperature down to 4 K.[7] (**c**) Decay curves recorded between 60 and 80 K and with a repetition rate of 5 kHz. All measurements were conducted with 374 nm excitation, while the emission wavelength was at the peak of the emission spectra.

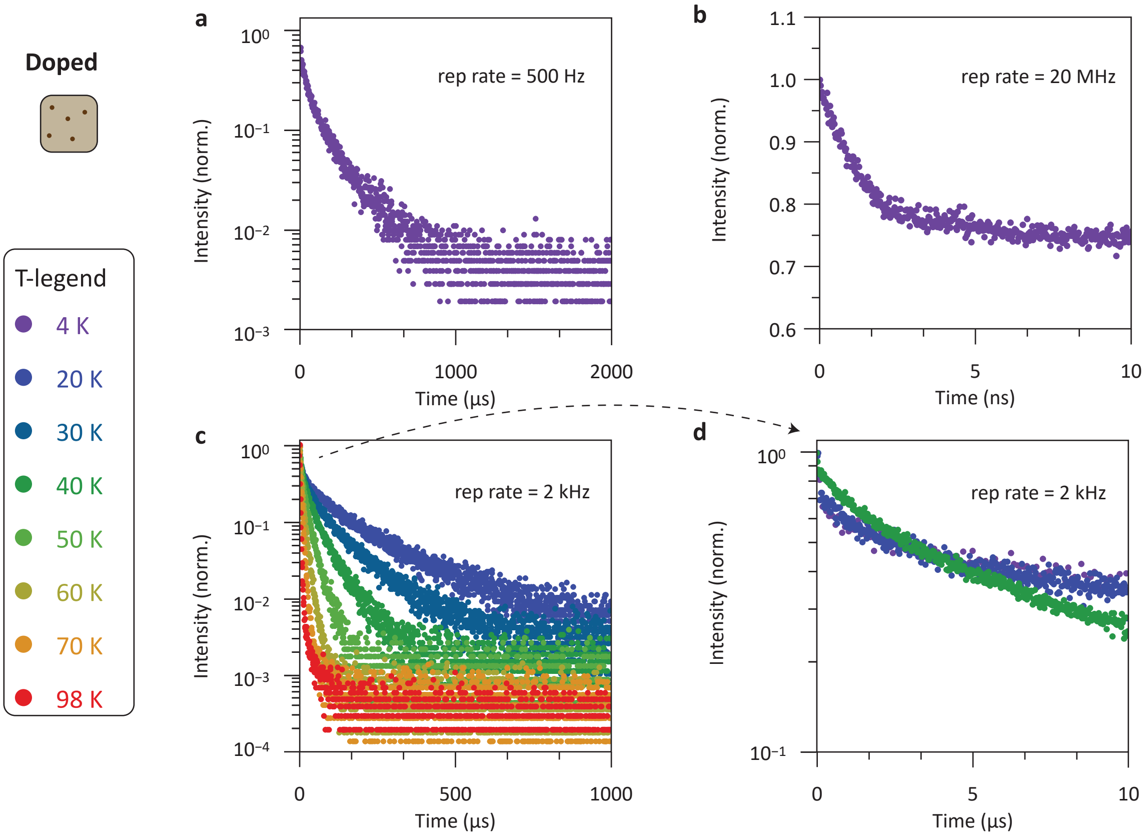

Figure S10 | Temperature-dependent luminescence decay curves of Yb^{3+}-doped $Cs_2AgBiBr_6$ NCs. (**a**) Decay curve recorded at 4 K, with a repetition rate of 500 Hz for excitation at 374 nm and emission at 690 nm. Note that, like for the undoped samples, on this timescale there is a fast component visible at the beginning of each decay curve. (**b**) Luminescence decay measurement with a faster laser (PicoQuant, λexc = 374 nm, pulse width = 54 ps, repetition rate 20 MHz) to capture the fast initial decay. (**c**) Overview of decay measurements recorded between 20 and 98 K, with a repetition rate of 2 kHz. (**d**) A zoom-in of the first 10 µs.

References

1. Creutz, S. E.; Crites, E. N.; De Siena, M. C.; Gamelin, D. R. Colloidal Nanocrystals of Lead-Free Double-Perovskite (Elpasolite) Semiconductors: Synthesis and Anion Exchange to Access New Materials. *Nano Lett.* **2018**, 18 (2), 1118–1123.

2. Makuła, P.; Pacia, M.; Macyk, W. How to Correctly Determine the Band Gap Energy of Modified Semiconductor Photocatalysts Based on UV–Vis Spectra. *J. Phys. Chem. Lett.* **2018**, pp. 6814–6817.

3. Tonti, D.; Van Mourik, F.; Chergui, M. On the Excitation Wavelength Dependence of the Luminescence Yield of Colloidal CdSe Quantum Dots. *Nano Lett.* **2004**, 4 (12), 2483–2487.

4. Roh, J. Y. D.; Smith, M. D.; Crane, M. J.; Biner, D.; Milstein, T. J.; Krämer, K. W.; Gamelin, D. R. Yb^{3+} Speciation and Energy-Transfer Dynamics in Quantum-Cutting Yb^{3+} -Doped $CsPbCl_3$ Perovskite Nanocrystals and Single Crystals. *Phys. Rev. Mater.* **2020**, 4 (10), 105405.

5. Shannon, R. D. Revised effective ionic radii and systematic studies of interatomic distances in halides and chalcogenides. *Acta Crystallogr. A* **1976**, 32 5, 751–767.

6. Tanner, P. A. Electronic Spectra of Yb^{3+} in Elpasolite Lattices. *Mol. Phys.* **1986**, 58 (2), 317–328.

7. Schade, L.; Wright, A. D.; Johnson, R. D.; Dollmann, M.; Wenger, B.; Nayak, P. K.; Prabhakaran, D.; Herz, L. M.; Nicholas, R.; Snaith, H. J.; Radaelli, P. G. Structural and Optical Properties of $Cs_2AgBiBr_6$ Double Perovskite. *ACS Energy Lett.* **2019**, 4 (1), 299–305.
